# The Impact of the National Nutrition Program 2017–2030 on People’s Food Purchases: A Revenue-Based Perspective

**DOI:** 10.3390/nu13093030

**Published:** 2021-08-30

**Authors:** Jianxiong Chen, Chung-Cheng Yang

**Affiliations:** Department of Accounting, National Yunlin University of Science and Technology, Yunlin 64002, Taiwan; d10825001@gemail.yuntech.edu.tw

**Keywords:** National Nutrition Program 2017–2030, food purchasing behavior, food policy, translog revenue function, revenue-based perspective

## Abstract

The General Office of the State Council of China promulgated the National Nutrition Program 2017–2030 in 2017 to guide the people to improve their food supply and nutritional intake. This study uses qualitative and quantitative information which are analyzed to estimate the change in people’s food purchases following the implementation of the National Nutrition Program 2017–2030, and puts forward measures that should be taken by the competent authorities and stakeholders. We use the translog revenue function of the food industry, and based on the data of listed companies of Chinese food enterprises from 2015 to 2020, and this study find that the National Nutrition Program 2017–2030 has had a positive impact on people’s food purchases, and the impact is more obvious in people’s food purchases from large food manufacturers. Finally, we also provide regulators with public policy implications, and provide food manufacturers with development suggestions.

## 1. Introduction

The purpose of this study is to explore the impact of China’s National Nutrition Program 2017–2030 (NNP) on people’s food purchases from the perspective of revenue. The World Declaration and Plan of Action on Nutrition in 1992 [1] are the basis of nutrition and food policies in many countries. In the nearly 30 years after the promulgation of the above-mentioned two policies, the Chinese government has promulgated many nutrition and food policies, and the physical quality and food supply of the population have been strongly improved [2]. With the progress of China’s economy, and the rapid development of the food industry and agriculture, China has increased the food supply for its population, which has improved the growth and development of the youth and has reduced malnutrition [3]. However, China still faces the challenge of malnutrition. Especially owing to insufficient food intake and/or poor quality, many areas still suffer from nutritional deficiencies.

Governmental policy can affect a country’s progress in achieving good nutrition and adequate food supplies. In 2017, the Chinese government promulgated the NNP, aiming at improving nutrition and the food supply at the national level, and formulated national nutrition and food strategies for 2020 and 2030 [4]. These policies have taken a series of major actions to help achieve the set goals; these include nutrition actions for the first 1000 days of life, actions to improve nutrition and food for students and the elderly, and nutrition and food interventions in rural areas. This has also been a major effort made by the Chinese government to improve the nutritional status and food access of the population, and it is possible these contribute to the sustainable developmental goals of “ending hunger, achieving food security and improving nutrition” of the United Nations [5].

China was once a developing country with the largest rural poor population in the world. Because of the various types and complex causes of poverty and the arduous task of antipoverty, China’s poverty problem has attracted broad attention from academics at home and abroad [6,7,8,9,10,11,12,13,14]. China still has a large rural poor population, and aging population in China is one of the main issues in poverty-stricken areas [15]. The poverty headcount ratio of the elderly in rural China was three times that in the urban areas [16]. The relationship between older people’s poverty and social exclusion in rural areas is complex, and the elderly are a significant poverty-afflicted group [17]. Many elderly people’s annual income is less than 1000 RMB [18]. In many rural areas, there are average annual incomes per person of a household ≤ 1000 RMB [19].

Nutrition problems can be roughly classified into two categories: insufficient food intake and unbalanced nutrition. That is, people may face food quantity problems and/or food quality problems [20]. At present, the nutritional problem of a considerable part of China’s population is the low purchasing power of citizens caused by poverty. In the contents of the National Nutrition Program 2017–2030, it is also mentioned that this policy will increase the support of the food supply for students, the elderly and populations in poverty-stricken areas. Therefore, this study focuses on the impact of the NNP on China’s actual situation of insufficient food supply for a considerable part of the population.

The promulgation of nutrition-related policies plays an important role in people’s food purchases and national health [21,22]. The NNP provides policy guidance and support for people’s food purchases. In view of the strong political and market influence of the Chinese government on citizens’ daily lives, we expect that this policy will prompt people to increase their spending on food purchases, that is, the NNP should have a positive impact on people’s food purchases.

With access to the food demand side (e.g., housewives, young mothers, the elderly) and the food supply side (e.g., supermarket managers), we obtained qualitative data for our analysis. We interviewed different groups of people affected by the NNP to explore the impact of the NNP on people’s food purchases. In addition, we also try to find the relationship between policy and people’s food purchases from the perspective of enterprise supply in economics. In this study, the impact of the NNP on people’s food purchases is discussed by constructing the translog revenue function of the food industry and using the annual pooling data of pure Chinese food enterprises from 2015 to 2020. The contributions of this study are as follows. First, as far as we know, we pioneered this kind of investigative reporting research, which uses a food industry revenue function in the research of people’s food purchase behavior to study the impact of policy. There is no existing literature on the impact of the NNP on people’s food purchases. This article will fill this gap in the literature. Second, according to our research, the COVID-19 pandemic, which broke out in early 2020, had a negative impact on the revenue of China’s food industry, which was enough to offset the increase in people’s intention for food purchases brought by the National Nutrition Program 2017–2030. We put forward specific measures for the food industry regulators, enterprises, investors and the public to consider. In the aforementioned ways, people can benefit more in the process of purchasing food. Third, under the current situation of frequent global trade, as one of the most influential countries in the world, the study of China’s nutrition and food policies will have a profound impact on the food production and policies of many countries. Our research can provide valuable references for other countries (especially those countries that have important food trade with China) in terms of nutrition policies, food trade policy formulation and adjustment of export food structures.

Section 2 of this study introduces the NNP. In Section 3, we provide the impact of the NNP on food consumers and food suppliers, and the background and hypothesis development are described in Section 4. In Section 5, we introduce the research methods and models. Section 6 and Section 7 are the results and conclusions respectively.

## 2. The National Nutrition Program 2017–2030

### 2.1. Main Objectives of the NNP

#### 2.1.1. Main Objectives to Be Achieved by 2020

By 2020, China’s nutrition regulatory system was basically improved; the nutrition work system was basically sound, and the nutrition work system at the provincial, city and county levels was gradually improved, and the nutrition work at the grass-roots level was strengthened. With the rapid development of the food and nutrition industries, food intake and nutrition services were increasingly abundant; the food supply of key groups was guaranteed, the malnutrition situation was significantly improved, and the population’s nutrition was significantly improved.

#### 2.1.2. Main Objectives to Be Achieved by 2030

By 2030, the goal is for the nutrition laws and regulations system to be improved, for the nutrition work system to be enhanced, for the food industry and nutrition industries to continue to develop, for the food intake and nutrition services to be more abundant, and for residents’ nutrition to be further improved.

### 2.2. Main Contents of the NNP

#### 2.2.1. For the 1000-Days Nutrition Action in Early Life

The goal is to carry out nutritional evaluations and food guidance before and during pregnancy. Thus, maternal and child health institutions should be promoted to provide nutritional guidance to pregnant women, and nutritional evaluations and food guidance should be included in pre-pregnancy and pregnancy examinations. This includes carrying out nutrition screening and food intervention for pregnant and postpartum women to reduce the rate of having low birth weight babies. It also establishes a nutrition consultation platform for the first 1000 days of life.

Included is the implementation of the maternal and child population nutrition intervention plan. It plans to continue to promote rural women’s supplementary nutrition, reduce the prevalence of anemia in pregnant women, prevent nutritional deficiencies in children, and increase food supply support. Special food support is to be provided to infants and young children of poor families.

It also strives to improve the rate of breastfeeding and cultivate scientific feeding behavior. Further, it sets out to improve the breastfeeding guarantee system, improve the breastfeeding environment, and set up maternity and infant rooms in public places, enterprises and institutions. It also is to support research and formulate scientific feeding strategies for infants and young children. Moreover, it intends to strengthen the monitoring of malnutrition cases in infants and young children, and to formulate and implement strategies for the prevention and control of foodborne diseases in infants and young children.

It also aims to improve the quality and safety level of infant food and promote the healthy development of the food industry. And it aims to strengthen the monitoring of infant formula food, supplementary food, food nutritional components and key pollutants, and it plans to make timely revisions and improve the standards of infant formula food and supplementary food. It also aims to improve food research and development (R&D) capacity and continuously improve the quality of infant formula food and supplementary foods.

#### 2.2.2. For Student Groups

The goal is to instruct students in food intake. It would encourage local governments to formulate recipe guides to meet the needs of students of different ages, guide students to eat nutritious meals, ensure the nutrition and food supply of students, and formulate and implement nutrition and food guidelines for meals.

It also would carry out food intake and nutrition guidance for students, as well as carry out nutrition education for students. In particular, the NNP has found it necessary to promote primary and secondary school students to strengthen nutrition education, and carry out various forms of nutrition education activities inside and outside classes in combination with the characteristics of students of different ages.

It would also adhere to the diversification of students’ food intake, and improve the ability and effect of food and nutrition support. And it would revise relevant national and industrial standards, improve the research and development capacity of various food technologies, and promote industrial development.

#### 2.2.3. For the Elderly

The goal is to monitor and evaluate the nutritional status and food intake of the elderly population. It would do this by relying on the national geriatric research institutes and primary medical and health care institutions, and by establishing and improving the nutrition screening and evaluation system for the elderly, compiling nutrition evaluation guidelines, and developing appropriate nutrition screening tools. Also, it would pilot the monitoring, screening and evaluation of the nutritional status of the elderly, and form a regional demonstration, gradually covering more than 80% of the elderly in the country, and basically mastering the nutritional status of the elderly.

It would also ensure the food supply for the elderly, and establish nutrition improvement measures to meet the needs of different elderly groups. It would accomplish this by relying on basic medical and health institutions, and by providing food guidance and consultation for home-based elderly. Also, it would develop foods suitable for the nutritional needs of the elderly. And it would give special nutrition and food intervention to the low-weight elderly, and gradually improve the overall nutritional level of the elderly population.

Lastly, it would establish a nutrition management and care system for the elderly. This would gradually bring the nutritional status of the elderly into the residents’ archives, so as to achieve seamless connection and effective management. And relying on the existing work foundation, it would include nutrition and food intake related work content in family health services. It would also promote the multi-sectoral cooperation mechanism and achieve the effective connection between nutrition and medical care.

#### 2.2.4. For Poverty-Stricken Areas

For poverty-stricken areas, the goal is to incorporate food and nutrition intervention into health poverty alleviation work, and carry out food and nutrition guidance according to local conditions. It would do this by piloting the monitoring of nutritional status, food consumption patterns and main nutritional components in food of various groups of people. It would formulate food and nutrition guidance programs according to local conditions, and carry out regionally accurate classification guidance and publicity. For poverty-stricken areas where residents still suffer from malnutrition, it would propose solutions and specific measures, and pilot them in areas where conditions permitted.

It would also continue to promote the implementation of the food and nutrition improvement plans for rural compulsory education students and the food and nutrition improvement project for children in poverty-stricken areas, and gradually cover all areas involved in the national poverty alleviation work.

It would also encourage schools in poverty-stricken areas to combine local resources and develop reasonable food guidance according to local conditions, and improve students’ eating conditions at school. Moreover, it would continue to monitor and evaluate the nutritional status and food safety risks of students in poverty-stricken areas. And according to the nutrition and food needs of people in poverty-stricken areas, it would formulate and improve relevant policies. It would lastly implement food and nutrition interventions for key populations in poverty-stricken areas to ensure their food supply.

### 2.3. Main Outcomes of the NNP

In May 2021, in the 7th “China National Nutrition Week”, Shanghai, one of the major cities in China, announced the completion status of the NNP objectives, as follows:

By 2020, all the phased targets had been achieved, including the following: basic food supply had been guaranteed for all people; the prevalence of anemia in children under 5 years old had dropped to 2.08%; the anemia rate for pregnant and lying-in women had dropped to 14.42%, which is lower than the 3.6% and 15% levels set by the government; the rate of exclusive breastfeeding for infants aged 0–6 months had increased to 62.93%; the growth retardation rate of children under 5 years old was controlled at 0.5%; the nutrition screening rate of inpatients and the treatment ratio of malnutrition inpatients increased to 70% and 35% respectively; the residents’ awareness rate of nutrition and health knowledge increased from 52.94% to 79.40%, and the awareness rates of nutrition knowledge of fruits and vegetables, beans and breastfeeding reached 83.59%, 47.90% and 48.81% respectively.

### 2.4. Time of the NNP Impact

Evidence shows that it takes about three years from policy implementation to the time change can be seen (e.g., the fiscal policy implemented by the Chinese central government in the first half of 1998, which greatly promoted China’s economic growth three years later [23]). Since the NNP is very significant with multiple benefits on household and individual basis, the interest lies on what immediate impact the implemented NNP has. Combined with the completion status of the NNP announced by the Shanghai Municipal Government in May 2021, we believe that this policy has had a rapid impact in China.

First, the Chinese government has a strong efficiency in policy implementation, which is different from that of most countries. China has only one ruling party, one ruling party state reflects strong policy implementation efficiency [24]. Taking the COVID-19 pandemic that broke out in early 2020 as an example, the Chinese government has demonstrated a strong response capability, and the COVID-19 pandemic has been effectively controlled through efficient policy implementation and high cooperation of the people with the government. We can expect that the implementation of the NNP 2017–2030, which is closely related to the Chinese people, will also be effectively implemented soon.

Second, the implementation status of the main policies of the Chinese government is related to the political achievements of the leaders of relevant departments of the provincial and municipal governments. Under the pressure from their superiors, the heads of food and health departments of local governments usually efficiently implement the policies uniformly deployed by the central government. The NNP was issued by the state Council of China. Under the guidance of the NNP, each local government has introduced detailed the NNP policy plans in various regions according to the specific local population’s nutrition, food intake and purchasing power. Examples of this are the Shanghai National Nutrition Plan (2019–2030) Implementation Plan, the Chongqing National Nutrition Plan (2017–2030) Implementation Plan, the Hainan National Nutrition Plan (2018–2030) Implementation Plan and the Shandong National Nutrition Plan (2018–2030), etc.

The above plans enable the NNP to be effectively implemented in different regions, which is more suitable for the actual situation of the region, and also greatly shortens the time for the policy to take effect.

Third, Shanghai announced in May 2021 the achievement of the 2020 NNP goals. From this fact, we can also see that the NNP has been implemented effectively in China, and the time from policy implementation to policy effectiveness is relatively short, which is consistent with the qualitative materials obtained from interviews with several interviewees.

## 3. The Impact of the NNP on Food Consumers and Food Suppliers

In order to provide important evidence to prove the changes experienced in food purchases and eating behaviors under the NNP, we conducted telephone interviews with housewives, part of the target population of the NNP (young mothers and the elderly) and supermarket managers.

### 3.1. The Impact of the NNP on Housewives

This study mainly investigates the impact of the NNP on consumers’ food purchases, so consumers’ perception of the NNP is crucial. In Chinese families, housewives are the main force in daily food purchases. In order to meet the daily dietary needs of families, housewives need to purchase many foods frequently, and they are sensitive to food prices and food purchase volume. Our interviews indicate that the principal benefit of the NNP to consumers is that consumers can buy more food at a lower expenditure, but consumers’ total expenditure on food has increased. In addition, the COVID-19 pandemic has caused great inconvenience to food purchases. Four housewives describe the impact of the NNP on their daily food purchases as follows:

“There are five people living together in my family, me, my children, my husband, and my father and mother. Food expenditures are one of the biggest expenses of my family. […] After the implementation of the NNP, one of my obvious feelings is that there are more promotional activities and price discounts on food in supermarkets. I spend less money on individual foods, but I used to hoard some food while taking advantage of discounts, which sometimes leads to that we can’t finish eating it all, so the total food expenditure of the family increased instead.”

(Interviewee 1)

“I am mainly responsible for purchasing food. My family is not rich, so I always have to budget carefully. I hope the family can save money on total expenses, including food expenses. However, after I saw the NNP content publicized by the community, I would rather reduce my spending on clothes and skin care products than the family’s food expenditures. […]”

(I2)

“[…] Although the COVID-19 pandemic in 2020 caused inconvenience to our going out, it also saved us a lot of money on food expenditures. […] Yeah, frankly speaking, our food purchasing did waste to a certain extent before, but during the COVID-19 pandemic, all the residents in my community were not allowed to go to the store to buy food, and only the community staff could carry out food distribution. The food delivered was just enough for our family’s consumption. […]”

(I3)

“After learning about the NNP on TV, I paid more and more attention to the health of my family. Now I often choose the expensive food to buy. In my opinion, the quality of the expensive food should be better than the cheap ones. […]”

(I4)

### 3.2. The Impact of the NNP on Young Mothers and Infants

Infants and young children are one of the main target groups of the NNP. Infants and young children are fed by breast milk by lactating women or milk powder by mothers. The implementation of the NNP is likely to benefit lactating women and infants. Two young mothers describe their changes in food purchases after the implementation of the NNP as follows:

“I’m glad that I learned the importance of trace elements and enriching blood through the NNP before I became a mother. I now have a ten-month-old baby, and I buy milk powder from Australia and New Zealand for her, which makes me feel more secure. I feel very happy watching her grow up healthily every day. […] I like to buy foreign food for my husband and parents now, and I feel that foreign food is safer. […] Paying more attention to family nutrition makes my family’s burden heavier and heavier. […]”

(I5)

“I buy everything for my children from abroad, the baby’s milk powder, clothes, bottles, toys I buy from abroad, which is very common in my company. In my impression, every young mother in my company treats her children like this. […] We must give the best to our children, which is our nature as mothers!”

(I6)

### 3.3. The Impact of the NNP on the Elderly

One of the contents proposed by the NNP is to “ensure the food supply for the elderly, and establish nutritional improvement measures to meet the needs of different elderly groups”. The elderly group is also one of the target groups of the NNP. Our interviews show that the implementation of the NNP promotes the elderly to pay more attention to their health, ensures the purchases of basic food for the poor elderly, and promotes the elderly with better financial conditions to purchase higher-quality and more expensive food, thus increasing part of the elderly’s expenditure on food. Three elderly people describe their experiences as follows:

“I’m too old to eat much now, but I think this: since I don’t eat much, I’ll try to eat better. […] the NNP was publicized in the village, and I heard from my neighbors that the country also advocates that the elderly should pay attention to nutrition, and now I try to buy expensive and good foods to eat. […] My children may be influenced by me. Now when they come to visit me, they always bring me supplements, but I don’t want them to spend money for me. I can still afford the money myself. […]”

(I7)

“After my death, the money can’t be taken away. Why should I keep it? […] After understanding the contents of the NNP, I became more determined about this idea. […] My wife and I now have a considerable pension every month. We eat well every month and never treat ourselves badly.”

(I8)

“I am 86 years old this year, and my children don’t give me a penny every month. […] I am very grateful to the government. In recent years, the government has given me 750 ¥ every month. […] Old friends and caring people in the village sometimes buy some rice, fish and meat for me, an elderly person living alone. The problem of eating has been solved, and I am satisfied.”

(I9)

### 3.4. The Impact of the NNP on Food Distributors

In addition to qualitative analysis of the impact of the NNP from the perspective of food consumers such as housewives, young mothers and the elderly, we also interviewed food suppliers. Due to the specific occupational environment, supermarket managers have a more comprehensive understanding of the impact of the NNP on food sales. A supermarket manager describes the changes in supermarket food sales as follows:

“[…] In the past few years after the implementation of the NNP, the fastest selling products on the shelf is food. We frequently restock the food. In addition to the frequent replenishment of imported food, the monthly shipment of low-priced food is also much higher than before the implementation of the NNP, which not only makes me to see the purchasing power of the middle class is rapidly improving, but also makes me feel that the food supply of the people below the middle class has been further improved. […] I hope the state can introduce more policies to ensure food supply. If the state can subsidize more food, it would be better. We are also willing to reduce food prices or pay a certain amount of profit to feed consumers.”

(I10)

Our qualitative analysis shows that the NNP makes people willing to pay more for food purchases. For poor people, the impact of the NNP on them is to help them ensure the supply of food, while for the non-poor people, the impact of the NNP on them is to pursue more expensive and higher-quality food.

## 4. Background and Hypothesis Development

In recent decades, managers and scholars have been interested in a series of impacts of policies. These include studies of the Organic Foods Production Act [25,26,27], the British Food Safety Act [28,29,30], the US Food Safety Modernization Act [31,32,33,34], EU food law [35,36,37,38], chain restaurant menu labeling [39,40,41,42], sugar-sweetened beverage labels [43,44,45,46], sugary drink tax [47,48], fat tax [49,50,51,52,53], and healthy food subsidies [54,55,56,57,58]. These papers focus on the impact of policies on society, public behavior, or industry.

One of the most influential food policies is the competitive food law. Over the past two decades, in view of the concern about the dietary status of students on campus, many states in the United States have formulated targeted policies to impose a series of restrictions on the food and beverages sold in schools, which are often referred to as “competitive foods” [59,60]. Further policy changes have been stimulated by the large increase in the obesity rates of students [61] and the evidence that competitive foods tend to include high-calorie foods and beverages such as sugary drinks and confectionery [62,63]. Scholars believe that with the increasing dependence of students on competitive foods, the supply of unhealthy competitive foods in schools also leads to the rapid increase of obesity [64]. In addition, more evidence suggests that the policy of setting nutrition standards for competitive foods promotes healthier students’ dietary intake, reduces the average weight of students, and improves the school food environment [65,66,67,68,69,70,71,72]. At present, the results of the states and regions that have implemented strong competitive food law show that this policy effectively reduces students’ intake of high-sugar foods and high-fat foods, and also reduces students’ dependence on these high-calorie foods [73,74,75,76,77,78,79]. Previous studies have shown that competitive food laws promote teenagers to buy healthier products [65] and maintain a healthier weight [71]. Moreover, competitive food laws also increase the service revenue of the food industry [80]. The significant impact of competitive food laws on people’s food purchases and on the food industry’s revenue in this developed market gives us a great drive to study the impact of the NNP, which was issued by an emerging market in 2017, on people’s food purchases and the food industry.

In recent years, one of the most influential food policies in China has been the NNP. The policy proposes to solve the problems of insufficient food and nutrition for some residents, and provides policy support to the food industry and agriculture. The NNP guarantees food and nutrition supplies at multiple levels. First, in terms of students’ food intake, the program encourages local governments to formulate recipe guides to meet the needs of students of different ages, so as to fully ensure students’ food intake. At the same time, one of the goals put forward by the program for student groups is to keep the growth retardation rate of rural primary and secondary school students below 5%, and reduce the height difference between urban and rural students. Second, in terms of food intake of residents in poor areas, the program proposes to incorporate food and nutrition intervention into health poverty alleviation work, carry out food intake guidance and support, and implement food and nutrition intervention for key groups in poverty-stricken areas to ensure their food supply. Third, in the aspect of infant food intake, the program puts forward 1000-days nutrition action in early life, which ensures infant nutrition and adequate food intake through policy support, and gives special food supply support to infants from poor families. Fourth, in the aspect of food intake of the elderly, various measures are taken to meet the dietary needs of the elderly, so as to alleviate the heavy burden of social public medical insurance caused by the increasing number of the elderly in China to a certain extent, and enhance the happiness of the “aging society”.

The Chinese government has a strong social appeal in social management and policy implementation. The above-mentioned policies of the program have been refined in various provinces and cities, and have been implemented rapidly. We expect that the implementation of the policy will encourage people to invest more in food. From the perspective of the supply side of food manufacturers, the increase of people’s purchases of food will be reflected in the increase in the sales quantity of food manufacturers’ products. In economics, enterprise revenue is product price multiplied by sales quantity. In a period of time, the price of food enterprises’ products will usually remain at a certain level. At this time, the quantity of products sold will have a direct impact on the revenue. From the perspective of the demand side, that is, the change of people’s purchase quantity of food will directly affect the revenue of food enterprises. In other words, the impact of the NNP on people’s food purchase quantities, from the perspective of enterprise supply side and financial figures, will be embodied in the change of revenue of the whole food industry. In summary, we expect that the NNP will increase people’s food purchases. In addition, advertising investment is closely related to product sales [81,82,83], large food manufacturers invest more in advertising, making their products more accessible to the public, thus gaining more trust from consumers. A large number of advertisements invested by large-scale food manufacturers have had a profound impact on people’s food purchases. The advertisements not only enrich the channels for people to understand food, but also the advantages of food promoted in the advertisements greatly expand people’s impression of food, stimulate people’s perception of food, and thus induce consumption desire, and as a result, we expect to see a much larger increase in purchases of food produced by large food manufacturers. Consequently, we propose the hypothesis of this study as follows:

**Hypothesis** **1.** *Ceteris paribus, the National Nutrition Program 2017–2030 has had a positive impact on people’s food purchases, and the impact is more obvious in people’s food purchases from large food manufacturers.*

## 5. Method

### 5.1. Theoretical Model

Chen and Yang [84] believes that the effective distribution of different types of employees is one of the prerequisites for the stable operation of enterprises. Food production enterprises in China generally have management personnel, R&D personnel and ordinary personnel to carry out various management, research and development, and business activities (e.g., the average number of R&D personnel in China’s food production enterprises from 2015 to 2020 is 152). The production function of food enterprises is as follows:

(1) $$y=f\left(x_{1},x_{2},x_{3},a\right)$$

*y* represents the sales of various food products carried out by the food enterprises; $x_{1},x_{2},x_{3}$ represent the amount of input from management personnel, R&D personnel and ordinary personnel respectively, and a represents the fixed asset input of the enterprise. The outputs and inputs must be nonnegative real numbers, i.e., y≥0, $x_{i}\geq 0$, a≥0, and *I =* 1, 2, 3; furthermore, the marginal product of the inputs in the production function has monotonically increasing and decreasing characteristics, i.e., $\partial f\left(\cdot \right)/\partial x_{i}\geq 0$,  ∂f(·)/∂a≥0, $\partial^{2}f\left(\cdot \right)/\partial x_{i}^{2}\leq 0$ and $\partial^{2}f\left(\cdot \right)/\partial a^{2}\leq 0$.

Food enterprises maximize their revenue at their existing technology level, and the corporate revenue function is as follows:

(2) $$r\left(p;x_{1},x_{2},x_{3},a\right)=max\;py\;subject\;to\;y=f\left(x_{1},x_{2},x_{3},a\right)$$

*r* represents the total revenue of food enterprises, and *p* represents the prices of foods provided by food enterprises.

General research uses the Cobb-Douglas function, and the corporate revenue function as follows:

(3) $$lnr=\alpha_{0}+\delta lnp+\overset{3}{\underset{i=1}{\sum }}\alpha_{i}lnx_{i}+\beta_{1}lna$$

Since the model has a single output, and the revenue function is characterized by homogeneous degree 1 in output prices (*δ* = 1), this study normalized by setting *p =* 1 [85]. Therefore, we can simplify equation (3) as follows:

(4) $$lnr=\alpha_{0}+\overset{3}{\underset{i=1}{\sum }}\alpha_{i}\;ln\;x_{i}+\beta_{1}ln\;a$$

Greene [86] believes that the optimal choice of the revenue function is a flexible function form, because the flexible function form can analyze the complex characteristics of the function, such as the second derivative and the elasticity of substitution. The most commonly used form of flexible functions by scholars is the translog model. A multiple input production is also usually estimated by the translog model [87,88]. Translog function can represent different levels of revenue elasticity [89]. Scholars have studied the use of translog revenue function in many industries [85,90]. The translog revenue function in this study is as follows [91]:

(5) $$lnr=\alpha_{0}+\overset{3}{\underset{i=1}{\sum }}\alpha_{i}\;ln\;x_{i}+\beta_{1}ln\;a+\frac{1}{2}\overset{3}{\underset{i=1}{\sum }}\overset{3}{\underset{l=1}{\sum }}\alpha_{il}\;ln\;x_{i}\;ln\;x_{l}+\frac{1}{2}\beta_{11}{\left(lna\right)}^{2}+\overset{3}{\underset{i=1}{\sum }}\gamma_{i1}lnx_{i}ln\;a$$

In the above equation, if $\alpha_{il}=\beta_{11}=\gamma_{i1}=0$, the translog model can be reduced to a Cobb–Douglas model. 

### 5.2. Data and Variables

#### 5.2.1. Data Source and Sample Period

We obtained the data for this study from the China Stock Market and Accounting Research (CSMAR) database (http://www.gtarsc.com/, accessed on 1 May 2021), which is available through the Wharton Research Data Services. CSMAR database was developed by Guo Tai An Company, which is the largest enterprise database in China, and is now widely used to obtain data of Chinese companies [92,93,94,95,96]. This database provides the data of variables in this study, such as the company’s revenue, the number of managers, the number of R&D personnel, the number of ordinary employees, and the ending balance of fixed assets, etc.

This study selected the data of Chinese food listed companies from 2015 to 2020. The National Nutrition Program 2017–2030 was implemented from 2017. In order to compare the difference of people’s food purchases before and after the implementation of the policy, the time span of the study data must be from before 2017 to after 2017. Taking 2017 when the National Nutrition Program 2017–2030 took effect, as the boundary, through the Chow Test, we analyzed the different food purchases of people influenced by the National Nutrition Program 2017–2030 before and after 2017.

Many previous studies have used similar methods. In order to study whether a firm’s IT investment in 1998 improved its productivity, Banker [97] took 1998 as the cut-off point and verified that the productivity of the firm in 1999 was higher than that in 1997 through regression analysis. Although Banker [97] clearly pointed out that the IT investment of the firm was carried out in 1998, in order to compare the productivity difference before and after IT investment, the time span of the data used in the study was from 1997 to 1999, and he collected the data before the event, which is consistent with us. Yang [98] studied the impact of the amendment of Taiwan’s certified public accountant act in 2007 on the public accounting industry. Taking 2007 as the boundary, he verified the significant positive impact of the amendment of the Taiwan’s certified public accountant act in 2007 on the public accounting industry through the translog model. Although the Taiwan’s certified public accountant act was amended in 2007, the study collected data from 1989 to 2017.

The data of this study consisted of 251 observations covering 6 fiscal years (Among them, 25 observations are large companies and 226 observations are non-large companies). We have excluded unreasonable observations, such as the number of employees or when the ending balance of fixed assets is zero. The deletion of the above data will make the research data closer to the actual situation of the food industry. In Table 1, we show the selection of samples.

#### 5.2.2. Variable Definitions

The theoretical model of this study is to explore the factors that influence people’s food purchases through economic theory and translog revenue function, but the actual market situation may not necessarily be consistent with the theory. Therefore, we need to verify the theory through the data of Chinese food manufacturers. In the next step, this study selects the corresponding proxy variables based on the theoretical variables proposed previously.

The dependent variable is the total revenue (*REVENUE*) of Chinese food enterprises. The revenue function is the maximization of profits under a given enterprise technology, so it can be seen that the revenue is the function of the input quantity and the output price [99]. In a period of time, the price of enterprises’ products will usually remain at a certain level, so this study normalized by setting *p* = 1 [85].

Human resource is one of the independent variables in this study. Wooldridge [100] pointed out that human resources and assets are the main inputs of translog revenue function, human resources play an important role in promoting the sustainable development of food enterprises [101]. Therefore, we use management personnel (*MANAGER*), R&D personnel (*RD*) and ordinary personnel (*ORDINARY*) as proxy variables of human resources. For the measurement of fixed assets, we use the ending balance of fixed assets (*FASSET*) as proxy variable. The above-mentioned *MANAGER*, *RD*, *ORDINARY* and *FASSET* are input quantities of the food industry.

The General Office of the State Council of China promulgated the NNP on 30 June 2017. We established the National Nutrition Program 2017–2030 (*POLICY*) as a dummy variable. When its value is 1, it indicates the years after the promulgation of the National Nutrition Program 2017–2030 (including 2018); if its value is 0, it means the years before the promulgation of the National Nutrition Program 2017–2030 (including 2017), which was used to test the impact of the promulgation of the National Nutrition Program 2017–2030 on the people’s food purchases. In addition, because most of the market shares are occupied by MALING, BRIGHT, MEIHUA, YILI and HAITIAN (the total revenue of the top five food manufacturers from 2015 to 2020 accounted for 69.75%, 67.05%, 67.18%, 63.61%, 60.99% and 59.55% of the total revenue of China’s food industry, respectively), and the fact that their foods are generally more trusted, China’s top five food manufacturers (*LARGE*) will be heavily influenced by the NNP. Therefore, we added a dummy variable, *LARGE*, which equals 1 if the enterprise is one of the big five enterprises. We bring together the definitions of the above variables in Table 2.

### 5.3. Estimation Model

This study uses translog revenue function to describe the correspondence between total revenue, human resources and investment in fixed assets of food enterprises, which is affected by *POLICY* and *LARGE*. Theoretical variables need to be transformed into proxy variables, thus, Equation (5) is rewritten as follows:

(6) $$\begin{array}{cc} lnREVENUE= & \alpha_{0}+\alpha_{1}lnMANAGER+\alpha_{2}lnRD+\alpha_{3}lnORDINARY+\beta_{1}lnFASSET\\ & +\frac{1}{2}\;\alpha_{11}\;{\left(ln\;MANAGER\right)}^{2}+\frac{1}{2}\;\alpha_{22}\;{\left(ln\;RD\right)}^{2}+\frac{1}{2}\;\alpha_{33}\;{\left(ln\;ORDINARY\right)}^{2}+\frac{1}{2}\;\beta_{11}\;{\left(ln\;FASSET\right)}^{2}\\ & +\alpha_{12}\;ln\;MANAGER\;ln\;RD+\alpha_{13}\;ln\;MANAGER\;ln\;ORDINARY+\alpha_{23}\;ln\;RD\;ln\;ORDINARY\\ & +\gamma_{11}\;ln\;MANAGER\;ln\;FASSET+\gamma_{21}\;ln\;RD\;ln\;FASSET+\gamma_{31}\;ln\;ORDINARY\;ln\;FASSET\\ & +\varphi_{1}\;LARGE+\varphi_{2}\;POLICY+\varphi_{3}\;LARGE\;POLICY \end{array}$$

The APE of variables on *REVENUE* can be expressed by the following equations:

The APE of *MANAGER* on *REVENUE*:

(7) $$\begin{array}{cc} \partial \hat{\;ln\;REVENUE}/ & \partial \;ln\;MANAGER\\ & ={\;\hat{\alpha }}_{1}+{\;\hat{\alpha }}_{11}\;\bar{ln\;MANAGER}+{\;\hat{\alpha }}_{12}\;\bar{ln\;RD}+{\;\hat{\alpha }}_{13}\;\bar{ln\;ORDINARY}\\ & +{\;\hat{\gamma }}_{11}\;\bar{ln\;FASSET} \end{array}$$

The APE of *RD* on *REVENUE:*

(8) $$\begin{array}{cc} \partial \hat{\;ln\;REVENUE}/ & \partial \;ln\;RD\\ & ={\;\hat{\alpha }}_{2}+{\;\hat{\alpha }}_{22}\;\bar{ln\;RD}+{\;\hat{\alpha }}_{12}\;\bar{ln\;MANAGER}+{\;\hat{\alpha }}_{23}\;\bar{lnORDINARY}\\ & +{\;\hat{\gamma }}_{21}\;\bar{ln\;FASSET} \end{array}$$

The APE of *ORDINARY* on *REVENUE*:

(9) $$\begin{array}{cc} \partial \hat{\;ln\;REVENUE}/ & \partial \;ln\;ORDINARY\\ & ={\;\hat{\alpha }}_{3}+{\;\hat{\alpha }}_{33}\;\bar{ln\;ORDINARY}+{\;\hat{\alpha }}_{13}\;\bar{ln\;MANAGER}+{\;\hat{\alpha }}_{23}\;\bar{ln\;RD}\\ & +{\;\hat{\gamma }}_{31}\;\bar{ln\;FASSET} \end{array}$$

The APE of *FASSET* on *REVENUE*:

(10) $$\begin{array}{cc} \partial \hat{\;ln\;REVENUE}/ & \partial \;ln\;FASSET\\ & =\;{\hat{\beta }}_{1}+{\;\hat{\beta }}_{11}\;\bar{ln\;FASSET}+{\;\hat{\gamma }}_{11}\;\bar{ln\;MANAGER}+{\;\hat{\gamma }}_{21}\;\bar{ln\;RD}\\ & +{\;\hat{\gamma }}_{31}\;\bar{ln\;ORDINARY} \end{array}$$

The APE of *LARGE* on *REVENUE*:

(11) $$\partial \hat{\;ln\;REVENUE}/\partial \;LARGE=\varphi_{1}+\varphi_{3}\;POLICY$$

The APE of *POLICY* on *REVENUE*:

(12) $$\partial \hat{\;ln\;REVENUE}/\partial \;POLICY=\varphi_{2}+\varphi_{3}\;LARGE$$

## 6. Results

### 6.1. Descriptive Statistics and Correlation Matrix

Table 3 shows the descriptive statistical results of the sample. The high standard deviation shows that the size and composition of China’s food enterprises are quite different. After the promulgation of the National Nutrition Program 2017–2030 in June 2017, with the increasing demand for food purchases, the average *REVENUE* of China’s food industry reached ¥5530 million in 2017, and in 2018, it reached the highest level in history. While there could be a consideration that this revenue comes from foreigners living in China, this consideration is set aside based on the following facts. The United Nations “National Immigration Stock Tendency” pointed out that the population of foreign nationals living in China in 2015 was 978 thousand, accounting for 0.07% of the Chinese population. In 2018, the number of foreigners in China was 705 thousand, and the proportion of foreigners in China’s population has declined [102]. This study does not consider the impact of foreigners on food consumption. First, the proportion of foreigners in China’s population has declined in recent years. Second, the proportion of foreigners in China’s population has never exceeded 0.1%, and the impact is negligible. However, in 2019 and 2020, the average revenue of China’s food industry declined, the possible reason is that as the promulgation time of this policy is getting farther and farther, the influence of the policy may decline to a certain extent. 

The average revenue of China’s food industry in 2020 is even lower than that before 2015. One factor that cannot be ignored is the COVID-19 pandemic which broke out in early 2020. Diseases have a significant negative impact on the economy [103]. For example, infectious diseases have dealt a heavy blow to per capita income and national GDP [104,105], human capital accumulation [106,107,108], house prices [109], etc. The long duration and wide impact of the COVID-19 pandemic have also prompted many scholars to conduct in-depth studies on the economic impact of the COVID-19 pandemic [110,111,112].

After the COVID-19 pandemic broke out in China, it caused panic among the people [113,114]. The government called on people to reduce going out and partying [115]. The closure of stores and restricted traffic have caused great inconvenience for people to buy food. In addition, studies have shown that whether in China [116], the United Kingdom [117], Scandinavian countries [118], or the United States [119], the COVID-19 pandemic has hindered consumption to a certain extent, and consumption has dropped rapidly. Obviously, consumers’ food expenditures have also been greatly affected.

However, we found that even in the context of the COVID-19 pandemic in 2020, the investment of Chinese food manufacturers in R&D personnel was still increasing. Chinese food production enterprises generally set up R&D departments [120]. R&D is the most critical determinant of a company’s productivity, growth, and competitive advantage [121,122]. With the development of the food industry, branding, differentiation, and personalization have increasingly become important factors for a new type of food to occupy the market, in which food research and development plays a vital role. For food production enterprises, every product has a life cycle, and even mature products must be improved according to market development. Product improvement and new product research and development cannot only extend the product cycle, but also broaden the market field of the company brand and create greater value. A successful new type of food often needs repeated market investigation, demonstration and experimentation, accumulating experience in repeated failures.

R&D expenditure plays a significant role in promoting sales volume [123,124,125]. In order to enhance competitiveness and promote people’s purchases of food, China’s food enterprises have continued to increase their investment in R&D personnel, and the average proportion of R&D personnel in the total employees (*EMPLOYEE*) has increased from 2.86% in 2015 to 4.10% in 2020. After the promulgation of the National Nutrition Program 2017–2030, the mean of R&D personnel in China’s food industry has also shown a trend of increasing year by year, which is conducive to food innovation and improvement of China’s healthy food structure, and is helpful to enhance people’s purchase intentions. However, on the whole, Chinese food enterprises’ investment in R&D personnel accounts for a low percentage of the total number of employees, which reflects that there is still a general shortage of investment in R&D personnel in the food industry.

We report Pearson and Spearman correlations between variables over the 6 years in Table 4. There is a significant positive correlation between *POLICY* and *REVENUE*, and there is also a significant positive correlation between *POLICY* and *RD*. Therefore, we can observe that the promulgation of the National Nutrition Program 2017–2030 has improved people’s intention for food purchase to a certain extent, which is partially reflected in the growth of the revenue of China’s food industry. In addition, after the National Nutrition Program 2017–2030 was promulgated, food enterprises also increased their investment in R&D personnel in order to meet the increasing food purchase demand of consumers.

### 6.2. The Impact of the National Nutrition Program 2017–2030 on People’s Food Purchases

This study mainly analyzes the impact of the National Nutrition Program 2017–2030 on people’s food purchases. Taking 2017, when the National Nutrition Program 2017–2030 took effect, as the boundary, through the Chow Test to analyze the revenue of food enterprises, the F statistics were 9.18. The rejection of the null hypothesis at a significant level of 1% indicates that the National Nutrition Program 2017–2030 would have an impact on people’s food purchases. Therefore, this study puts *POLICY* as a dummy variable into the model to further analyze its impact on the function.

### 6.3. Estimation Results of Revenue Function of Food Enterprises

#### 6.3.1. The Revenue Function

Table 5 shows the estimated results of the revenue function of Chinese food manufacturers. Instead of using the simple Cobb-Douglas format (Log-linear), this study uses the more flexible translog format. We needed to check whether the translog format can provide a correct representation of the revenue function of food manufacturers. Therefore, this study tests whether these conditions in Equation (6) are met:

(13) $$\alpha_{il}=\beta_{11}=\gamma_{i1}=0\;\mathrm{for}\;\mathrm{all}\;i=1,2,3.$$

Table 5 shows that the result of the F statistical value is 3.87, which significantly rejects the null hypothesis of the log-linear specification, indicating that the translog format is suitable for analyzing the revenue function of Chinese food manufacturers. 

#### 6.3.2. People’s Food Purchases

Table 6 shows the APE of different variables in this study on *REVENUE*. It can be seen from Table 6 that the APE of *POLICY* to the total revenue of food enterprises was positive and significant. At the same time, Table 5 shows that the estimated value of the *POLICY* coefficient was significantly positive in the revenue function equation. These results showed that the National Nutrition Program 2017–2030 increased people’s food purchases. We believe this might have been mainly due to the positive impact of the National Nutrition Program’s 2017–2030 support for food supply on people’s food purchases. Furthermore, from Table 6, it can be seen that the APE of *POLICY* on *LARGE* revenue was 0.114 and significant, which is greater than 0.111 for non-*LARGE*, indicating that the National Nutrition Program 2017–2030’s positive impact on people’s food purchases is more obvious when people buy food from *LARGE*. This is because *LARGE* occupies a larger market share, and *LARGE* foods are usually more trusted by consumers. This verified Hypothesis 1.

In addition to testing the APE of *POLICY* and *LARGE* on the revenue of food enterprises, this study also investigates the impact of human resources and fixed assets on people’s food purchases. Hosseininia and Ramezani [101] indicated that human resources are one of the important factors for the sustainable development of food enterprises. Table 6 shows that in terms of human resources, the APE of *MANAGER*, *RD* and *ORDINARY* for *REVENUE* is positive and significant. This indicates that the investment of food enterprises on employees had a positive impact on people’s food purchases. In addition, Table 6 also shows that the APE of *FASSET* is positive and significant for *REVENUE*, which indicates that the increase of fixed assets investment of food manufacturers also had a positive impact on people’s food purchases. We believe that the possible reason is that with the increase in the number of employees of various types as well as the increase of fixed asset investment, food manufacturers are becoming bigger and stronger. In particular, the increasing number of R&D personnel from 2015 to 2020 has led to more research and development of new foods. This kind of food is usually more popular in the market, which stimulates people’s stronger desire to buy food. Therefore, the *MANAGER*, *RD*, *ORDINARY* and *FASSET* invested by food manufacturers promoted people’s food purchases.

## 7. Conclusions and Discussion

We use the translog revenue function to estimate the impact of the National Nutrition Program 2017–2030 on people’s food purchases, and use an annual pooling data of food enterprises for the period 2015–2020 to make an empirical analysis. This research is the first to use a food industry revenue function to study the impact of policy on food purchase behavior. The research results show that the National Nutrition Program 2017–2030 has had a positive impact on people’s food purchases, and the impact is more obvious in people’s food purchases from large food manufacturers. However, it is worth noting that although our research results show that the National Nutrition Program 2017–2030 has had a positive impact on people’s food purchases, the overall revenue of the food industry has continued to decline since 2019, and the possible reason is that as the promulgation time of this policy is getting farther and farther, the influence of the policy may decline to a certain extent. In addition, the COVID-19 pandemic, which broke out in early 2020, also had a significant negative impact on the revenue of China’s food industry, which was enough to offset the increase in people’s intention for food purchases brought by the National Nutrition Program 2017–2030.

Our results show that the NNP has a positive impact on people’s food purchases. However, the sharp decline in the revenue of China’s food industry in 2020 caused by the COVID-19 pandemic requires the management authorities to reflect. In the face of major public health events, the NNP may need to be revised synchronously to cope with the adverse impact of major public health events, so that people can still have sufficient food supplies even when it is inconvenient to go out and make purchases. For Chinese food manufacturers, they should conscientiously fulfill the requirements of the latest food and nutrition policies with a responsible attitude toward consumers, and increase the investment of R&D personnel, so as to increase the attraction of their products to consumers through healthy and diversified products. In addition to the competent authorities, investors and the public are also powerful supervisors of food policy implementation. In addition to naturally favoring high-return food enterprises, investors should also have a long-term perspective, allowing capital to flow to areas that are suitable for the food needs of the Chinese people in the long term.

The limitation of this study is that although we consider the investment in human resources and fixed assets (*MANAGER*, *RD*, *ORDINARY*, *FASSET*), for other potential factors, such as production technology, R&D capital, and the possible significant impact of the NNP on the improvement of food quality, it is difficult to obtain these data, so this study cannot consider these potential factors. We pay special attention to the impact of the NNP on the improvement of food quality, and if data from this perspective can be obtained in the future, it may be the direction of further research. 

## Figures and Tables

**Table 1:** Sample selection for this study.

Total companies on CSMAR from 2015–2020	366
Deletions:	
(A) Zero whole–year revenue	(39)
(B) Zero management personnel	(19)
(C) Zero research and development personnel	(14)
(D) Zero ordinary personnel	(16)
(E) Zero ending balance of fixed assets	(27)
Final companies on CSMAR from 2015–2020	251

**Table 2:** Variable definitions.

Variable		Definition
Theoretical Variable	Proxy Variable	
*r*	*REVENUE*	Total revenue of food enterprises, expressed in million ¥
*x_1_*	*MANAGER*	Total number of management personnel
*x_2_*	*RD*	Total number of research and development personnel
*x_3_*	*ORDINARY*	Total number of ordinary personnel
	*EMPLOYEE*	Total number of employees
*a*	*FASSET*	Total ending balance of fixed assets
	*LARGE*	A dummy variable that equals one if the manufacturer is one of China’s top five food manufacturers, and 0 otherwise
	*POLICY*	A dummy variable that equals one if the year is one of the years after 2017, and 0 otherwise

**Table 3:** Descriptive statistics.

**Panel A:**	**2015 (*n* = 30)**					**Panel B:**	**2016 (*n* = 35)**				
**Variables**	**Mean**	**Median**	**Max**	**Min**	**Std. Dev.**	**Variables**	**Mean**	**Median**	**Max**	**Min**	**Std. Dev.**
*REVENUE*	¥5480.00	¥1860.00	¥59,900.00	¥151.00	¥11,200.00	*REVENUE*	¥5020.00	¥2150.00	¥60,300.00	¥259.00	¥10,600.00
*MANAGER*	6.43	6.00	12.00	4.00	1.76	*MANAGER*	5.74	5.00	8.00	3.00	1.56
*RD*	149.00	90.50	703.00	7.00	147.44	*RD*	140.20	76.00	588.00	7.00	142.47
*ORDINARY*	5210.70	2561.50	57,617.00	278.00	10,270.44	*ORDINARY*	4516.11	2332.00	54,632.00	303.00	9097.12
*EMPLOYEE*	5366.13	2776.00	57,971.00	328.00	10,306.92	*EMPLOYEE*	4662.06	2494.00	54,983.00	336.00	9142.06
*FASSET*	¥1980.00	¥809.00	¥14,600.00	¥48.14	¥3240.00	*FASSET*	¥1780.00	¥768.00	¥13,100.00	¥42.51	¥2860.00
**Panel C:**	**2017 (*n* = 37)**					**Panel D:**	**2018 (*n* = 41)**				
**Variables**	**Mean**	**Median**	**Max**	**Min**	**Std. Dev.**	**Variables**	**Mean**	**Median**	**Max**	**Min**	**Std. Dev.**
*REVENUE*	¥5530.00	¥2190.00	¥67,500.00	¥254.00	¥11,700.00	*REVENUE*	¥5820.00	¥2340.00	¥79,000.00	¥319.00	¥12,800.00
*MANAGER*	5.81	5.00	9.00	3.00	1.58	*MANAGER*	5.66	6.00	8.00	3.00	1.49
*RD*	137.32	86.00	403.00	6.00	120.50	*RD*	158.05	93.00	557.00	11.00	151.71
*ORDINARY*	4407.30	2402.00	53,185.00	289.00	8645.12	*ORDINARY*	4745.02	2228.00	55,710.00	322.00	8721.64
*EMPLOYEE*	4550.43	2510.00	53,531.00	301.00	8685.40	*EMPLOYEE*	4908.73	2419.00	56,079.00	359.00	8762.55
*FASSET*	¥1750.00	¥793.00	¥13,300.00	¥69.99	¥2770.00	*FASSET*	¥1760.00	¥916.00	¥14700.00	¥68.05	¥2750.00
**Panel E:**	**2019 (*n* = 50)**					**Panel F:**	**2020 (*n* = 58)**				
**Variables**	**Mean**	**Median**	**Max**	**Min**	**Std. Dev.**	**Variables**	**Mean**	**Median**	**Max**	**Min**	**Std. Dev.**
*REVENUE*	¥5590.00	¥2060.00	¥90,000.00	¥234.00	¥13,300.00	*REVENUE*	¥5370.00	¥2020.00	¥96,500.00	¥124.00	¥13,400.00
*MANAGER*	5.70	5.00	12.00	3.00	1.88	*MANAGER*	5.78	6.00	11.00	1.00	1.90
*RD*	160.48	103.50	782.00	7.00	166.86	*RD*	168.45	122.00	820.00	6.00	170.53
*ORDINARY*	4258.06	2176.50	58,636.00	275.00	8372.54	*ORDINARY*	4101.79	2178.00	58,702.00	135.00	7944.95
*EMPLOYEE*	4424.24	2335.00	59,052.00	318.00	8419.16	*EMPLOYEE*	4276.02	2309.50	59,159.00	172.00	7999.23
*FASSET*	¥1820.00	¥849.00	¥18,300.00	¥46.59	¥3090.00	*FASSET*	¥1780.00	¥849.00	¥23,300.00	¥49.01	¥3490.00

**Table 4:** Correlation matrix for dependent and independent variables (*p*-values in parentheses).

	REVENUE	MANAGER	RD	ORDINARY	EMPLOYEE	FASSET	LARGE	POLICY
**REVENUE**	1.000	0.120	0.481	0.864	0.871	0.808	0.562	0.024
	-----	(0.058)	(0.000)	(0.000)	(0.000)	(0.000)	(0.000)	(0.070)
**MANAGER**	−0.054	1.000	0.043	0.086	0.085	0.092	−0.026	−0.072
	(0.394)	-----	(0.499)	(0.175)	(0.181)	(0.146)	(0.687)	(0.258)
**RD**	0.328	0.044	1.000	0.359	0.404	0.422	0.271	0.057
	(0.000)	(0.484)	-----	(0.000)	(0.000)	(0.000)	(0.000)	(0.036)
**ORDINARY**	0.944	−0.074	0.281	1.000	0.998	0.776	0.498	−0.021
	(0.000)	(0.240)	(0.000)	-----	(0.000)	(0.000)	(0.000)	(0.743)
**EMPLOYEE**	0.945	−0.073	0.297	1.000	1.000	0.783	0.499	−0.018
	(0.000)	(0.249)	(0.000)	(0.000)	-----	(0.000)	(0.000)	(0.773)
**FASSET**	0.884	−0.036	0.297	0.858	0.859	1.000	0.538	−0.001
	(0.000)	(0.565)	(0.000)	(0.000)	(0.000)	-----	(0.000)	(0.985)
**LARGE**	0.714	−0.019	0.249	0.568	0.570	0.749	1.000	−0.070
	(0.000)	(0.768)	(0.000)	(0.000)	(0.000)	(0.000)	-----	(0.268)
**POLICY**	0.009	−0.072	0.068	−0.020	−0.019	−0.007	−0.070	1.000
	(0.089)	(0.255)	(0.028)	(0.754)	(0.769)	(0.913)	(0.268)	-----

**Table 5:** Estimation of translog revenue function for data pooled over 2015–2020 (*t*–statistics in parentheses).

$\begin{array}{cc} lnREVENUE= & \alpha_{0}+\alpha_{1}lnMANAGER+\alpha_{2}lnRD+\alpha_{3}lnORDINARY+\beta_{1}lnFASSET+\frac{1}{2}\;\alpha_{11}\;{\left(ln\;MANAGER\right)}^{2}\\ & +\frac{1}{2}\;\alpha_{22}\;{\left(ln\;RD\right)}^{2}+\frac{1}{2}\;\alpha_{33}\;{\left(ln\;ORDINARY\right)}^{2}+\frac{1}{2}\;\beta_{11}\;{\left(ln\;FASSET\right)}^{2}+\alpha_{12}\;ln\;MANAGER\;ln\;RD\\ & +\alpha_{13}\;ln\;MANAGER\;ln\;ORDINARY+\alpha_{23}\;ln\;RD\;ln\;ORDINARY+\gamma_{11}\;ln\;MANAGER\;ln\;FASSET\\ & +\gamma_{21}\;ln\;RD\;ln\;FASSET+\gamma_{31}\;ln\;ORDINARY\;ln\;FASSET+\varphi_{1}\;LARGE+\varphi_{2}\;POLICY\\ & +\varphi_{3}\;LARGE\;POLICY \end{array}$			
Variables	Coefficient	Variables	Coefficient
	*t*-Statistic		*t*-Statistic
Intercept	−4.573	(ln*MANAGER*)(ln*RD*)	0.072
	(−0.468)		(0.626)
ln*MANAGER*	2.984	(ln*MANAGER*)(ln*ORDINARY*)	0.813 ***
	(1.371)		(4.720)
ln*RD*	1.039 *	(ln*RD*)(ln*ORDINARY*)	−0.530 ***
	(1.685)		(−3.363)
ln*ORDINARY*	−2.573 **	(ln*MANAGER*)(ln*FASSET*)	−0.043
	(−2.507)		(−0.890)
ln*FASSET*	2.597 **	(ln*RD*)(ln*FASSET*)	−0.075 *
	(2.142)		(−1.824)
(ln*MANAGER*) ^2^	0.387 *	(ln*ORDINARY*)(ln*FASSET*)	0.128 *
	(1.751)		(1.889)
(ln*RD*) ^2^	0.092 ***	*LARGE*	1.238 ***
	(3.382)		(8.229)
(ln*ORDINARY*) ^2^	−0.046	*POLICY*	0.111 **
	(−1.143)		(2.013)
(ln*FASSET*) ^2^	−0.053	*LARGEPOLICY*	0.003
	(−1.361)		(0.019)
Adjusted R–squared	0.899		
System degrees of freedom	251		
*Test of log–linear specification* ($H_{0}:\;\alpha_{il}=\beta_{11}=\gamma_{i1}=0;\;i=1,\;2,\;3$)			
*F*–statistic	3.87		
Significance level	0.000		

Note: ***, **, * Denotes significantly difference from zero at the 1%, 5%, and 10% levels, respectively (two–tailed test). The model was estimated using ordinary least squares. All variables are as defined in Table 2.

**Table 6:** APE of human resources, *FASSET*, *LARGE*, and *POLICY* on revenue.

APE	APE Estimated Value	Significance Test
		$H_{0}:{\;\hat{\alpha }}_{1}={\;\hat{\alpha }}_{11}={\;\hat{\alpha }}_{12}={\;\hat{\alpha }}_{13}={\;\hat{\gamma }}_{11}=0$
*APE_MANAGER*	0.158	*F*–statistic = 6.05
		Significance level = 0.00
		$H_{0}:{\;\hat{\alpha }}_{2}={\;\hat{\alpha }}_{22}={\;\hat{\alpha }}_{12}={\;\hat{\alpha }}_{23}={\;\hat{\gamma }}_{21}=0$
*APE_RD*	0.141	*F*–statistic = 3.07
		Significance level = 0.02
		$H_{0}:{\;\hat{\alpha }}_{3}={\;\hat{\alpha }}_{33}={\;\hat{\alpha }}_{13}={\;\hat{\alpha }}_{23}={\;\hat{\gamma }}_{31}=0$
*APE_ORDINARY*	0.701	*F*–statistic = 6.76
		Significance level = 0.00
		$H_{0}:{\hat{\beta }}_{1}={\hat{\beta }}_{11}={\;\hat{\gamma }}_{11}={\;\hat{\gamma }}_{21}={\;\hat{\gamma }}_{31}=0$
*APE_FASSET*	0.069	*F*–statistic = 4.06
		Significance level = 0.00
*APE_LARGE*		$H_{0}:{\hat{\varphi }}_{1}={\hat{\varphi }}_{3}=0$
When *POLICY* = 0	1.238	*F*–statistic = 21.53
When *POLICY* = 1	1.241	Significance level = 0.00
*APE_POLICY*		$H_{0}:{\hat{\varphi }}_{2}={\hat{\varphi }}_{3}=0$
When *LARGE* = 0	0.111	*F*–statistic = 2.33
When *LARGE* = 1	0.114	Significance level = 0.09

## Data Availability

All relevant data are within the manuscript.
